# The Prognostic Significance of Bronchoalveolar Lavage Cellular Analysis in Evaluating Disease Burden in Non-Cystic Fibrosis Bronchiectasis

**DOI:** 10.3390/life16020206

**Published:** 2026-01-27

**Authors:** Ahmet Yurttaş, Deniz Çelik, Sertan Bulut, Özkan Yetkin, Hüseyin Lakadamyalı

**Affiliations:** 1 Faculty of Medicine, Department of Pulmonology, Alanya Alaaddin Keykubat University, 07425 Alanya, Turkey; deniz.celik@alanya.edu.tr (D.Ç.); ozkan.yetkin@alanya.edu.tr (Ö.Y.); huseyin.lakadamyali@alanya.edu.tr (H.L.); 2 Ankara Atatürk Sanatorium Training and Research Hospital, 06290 Ankara, Turkey; drsertanbulut@hotmail.com

**Keywords:** bronchiectasis, bronchoalveolar lavage, eosinophils, alveolar macrophages

## Abstract

Objective: This study aimed to investigate the relationship between bronchoalveolar lavage (BAL) cellular profiles, microbiological status, and clinical outcomes such as hospital admission in adult patients with non-cystic fibrosis bronchiectasis. Methods: A retrospective cross-sectional study was conducted on thirty adult bronchiectasis patients. Demographic, clinical, and laboratory data were collected. The cellular components of BAL fluid (macrophages, neutrophils, lymphocytes, and eosinophils) were analyzed. Patients were grouped according to the presence of microbial culture growth and history of hospitalization in the past year. Statistical analyses were performed to determine significant relationships. Results: The median age was 57 years, and the gender distribution was equal. There was no significant difference in BAL cellular profiles between groups with and without culture growth. However, in the group with a hospital admission in the past year, BAL showed a significantly lower percentage of alveolar macrophages (20% vs. 47%, *p* = 0.011) and a higher percentage of eosinophils (5% vs. 1%, *p* = 0.036). The hospitalized group also showed a trend toward a higher neutrophil percentage and a lower lymphocyte/neutrophil ratio. Furthermore, surprising associations were noted, such as a higher BAL macrophage count in married individuals and higher BAL eosinophilia in patients with diabetes. Conclusions: BAL cellular analysis provides valuable information beyond routine microbiological investigations in bronchiectasis. The low-alveolar-macrophage and high-eosinophil profile was found to be significantly associated with hospitalization, and this profile has the potential to serve as a prognostic biomarker in defining the “high-risk” phenotype. These findings highlight the complexity of the local inflammatory response and reveal the potential role of BAL in developing personalized treatment strategies for patients with bronchiectasis.

## 1. Introduction

Bronchiectasis is defined radiographically as permanent dilation of the bronchi and clinically as cough, sputum production, and recurrent chest infections [1]. Abnormal and permanent dilation of the bronchi occurs because of damage to the elastic and muscular structures of the bronchial wall. In 2013, the prevalence of bronchiectasis without cystic fibrosis in the United States was reported to be 139 per 100,000 [2]. According to the same data, the prevalence of bronchiectasis increased by approximately 40% compared to 2003, reaching a significant level of 566 per 100,000 people [1].

Among the etiological factors contributing to the development of bronchiectasis, infections are commonly reported as the primary cause; however, the underlying cause and microbiome may vary significantly across different regions of the world [3]. While the vicious cycle of infection and inflammation is widely accepted as the pathogenic model of bronchiectasis, a newer paradigm suggests that the quartet of inflammatory responses (acute and chronic airway infection, bronchiectasis/lung damage, and airway epithelial cell and ciliary dysfunction) interact in a complex manner, ultimately forming a “vicious vortex” that triggers the development and clinical manifestations of bronchiectasis [4]. Progress in translational research has enhanced comprehension of the disease’s pathobiology, particularly regarding airway inflammation, microbiome dynamics, and mucociliary dysfunction.

In patients with localized bronchiectasis without a clear preceding acute event, bronchoscopy should be considered to rule out the possibility of anatomical partial airway obstruction (e.g., tumor or foreign body) [4]. Because bronchiectasis patients also have compromised lung microbiota, bronchoalveolar lavage (BAL) and bronchoalveolar lavage fluid (BALF) obtained via bronchoscopy can provide information about this compromise. BAL has become a widely used diagnostic tool for pulmonary diseases. Obtaining samples from the lower respiratory tract via BAL allows for the collection of separable airway and alveolar cells, as well as non-cellular soluble proteins and other biomolecules, making it an important research tool for examining both healthy and diseased lungs [5]. Normally, BALF samples obtained from healthy, non-smoking control groups contain 80–90% alveolar macrophages (AMs), 5–15% lymphocytes, 1–3% polymorphonuclear neutrophils (PMNs), <1% eosinophils, and <1% mast cells [6,7].

Neutrophils play an important role in the development and progression of bronchiectasis. Neutrophilic infiltration has been reported in biopsies taken from the bronchial mucosa of patients [8]. In addition, an increase in blood neutrophils, which correlates with the severity of the disease and bacterial colonization, has been reported in patients with bronchiectasis [9]. The lymphocyte–neutrophil ratio is used as a biological marker of inflammation. It is calculated by dividing the number of neutrophils obtained from a peripheral blood sample by the number of lymphocytes. The lymphocyte–neutrophil ratio has been used as a biomarker in many diseases because the physiological response of circulating leukocytes to inflammation is an increase in circulating neutrophils and a decrease in lymphocyte count [10].

This study examined the BALF changes, cell distributions, proliferation, underlying disease, culture results, inflammation markers, and marital status in patients with non-cystic bronchiectasis.

## 2. Method

This retrospective cross-sectional cohort study is based on the analysis of records of adult patients (≥18 years) diagnosed with bronchiectasis and followed up and treated at Alanya Training and Research Hospital between January 2024 and June 2025. Patients were identified using the hospital’s information system through ICD-10 codes and clinical records. The study protocol was approved by the Ethics Committee of Alanya Alaaddin Keykubat University (10.12.2025-18-1).

Patients’ demographic characteristics (age, gender, body mass index, smoking history), comorbidities (hypertension, diabetes, heart failure, asthma, COPD, etc.), clinical symptoms (shortness of breath, cough, sputum, hemoptysis, weight loss, etc.), and physical examination findings (clubbing, crackles, etc.) were recorded using a standardized data collection form.

Laboratory parameters such as complete blood count, biochemical tests (urea, creatinine, albumin), and acute phase reactants (CRPs) were examined. High-resolution chest computed tomography (HRCT) images of all patients were evaluated by a radiologist for the presence, type (tubular, cystic, traction), and extent (number of affected lobes) of bronchiectasis. Respiratory function tests (RFTs) were performed, and FEV_1_, FVC, and DLCO values were recorded.

The FACED score was used to classify the severity of the disease [it consists of the parameters FEV_1_, age, chronic *Pseudomonas aeruginosa* colonization, radiological extent, and degree of dyspnea (mMRC)]. The frequency of exacerbations was assessed by the number of hospital admissions or hospitalizations in the last year. Mild exacerbations that did not require hospital admissions were excluded from analysis. The study excluded patients under the age of eighteen, those with impaired consciousness, those with Alzheimer’s disease, those with dementia, those with Parkinson’s disease, those with active cancer, those with mild infectious attacks not requiring hospitalization, patients with severe hypoxemia, and patients for whom fiberoptic bronchoscopy was contraindicated.

The growth of pathogenic microorganisms in sputum culture was considered “colonization/infection”. The BALF samples were sent to the microbiology laboratory for bacterial, fungal, and direct microscopic examination and culture for tuberculosis. The samples were inoculated onto blood agar, chocolate agar, eosin–methylene blue agar, and Sabouraud agar. The samples were stained using the Ziehl–Neelsen staining method and inoculated onto a Löwenstein–Jensen medium for tuberculosis. The BALF samples taken for cell counting were sent to the laboratory within 30 min. The samples were counted using the Sysmex XN-1500 (Hamburg, Germany) device in body fluid mode. When the concentration was very high, the accuracy of the results obtained by the machine was confirmed by preparing a cellpack diluent and confirming it with a Thoma. The cellular analysis of BALF obtained by bronchoscopy (percentages of alveolar macrophages, neutrophils, lymphocytes, and eosinophils) was recorded, and the ratios of these cells (lymphocytes/neutrophils, macrophages/lymphocytes, etc.) were calculated.

The Kolmogorov–Smirnov or Shapiro–Wilk test was used for the distribution of all numerical values. Categorical data were evaluated with the chi-square or Fisher’s exact test, as appropriate, and numerical data with Student’s *t*-test or the Mann–Whitney U test. *p* values below <0.05 in our study were considered statistically significant. Analyses were performed using the International Business Machines Statistical Package for the Social Sciences (IBM SPSS) 22 program.

## 3. Results

The study included a total of 30 patients with bronchiectasis. The mean age of the participants was 55.36 years (±14.33 standard deviation). The gender distribution was equal (50% female, 50% male). The average Body Mass Index was 27.09 (min–max, 18.51–37.10). It was observed that, out of 30 patients, the number of cases with a Body Mass Index > 25 was 19. The patients’ smoking status was as follows: non-smokers, 13 (43.3%); smokers, 17 (56.7%). The patients’ comorbidities are as follows: COPD in 9 (30%), hypertension in 8 (26.7%), diabetes mellitus in 4 (13.3%), coronary syndrome in 5 (16.7%), and asthma in 1 (3.3%). Seventy percent of the patients were married. The distributions of the clinical symptoms, physical examination findings, chest CT findings, and bronchiectasis scores are summarized in Table 1 and Table 2.

Nearly all patients (24 patients, 80%) had a history of infection. The most common symptoms were cough and sputum production, followed by shortness of breath. One-third of cases had intermittent hemoptysis. Clubbing was the most common finding in the physical examination, present in 43.4% of cases.

Complete blood counts, renal and hepatic function tests, and C-reactive protein values are shown in Table 3.

Respiratory function tests revealed a median FVC of 72% (25–108), a median FEV_1_ of 68% (18–110), and a median FEV_1_/FVC ratio of 76% (39–92), indicating varying degrees of obstructive impairment in patients (Table 3).

BALF cellular components were compared between groups with and without culture growth. Analysis of data from a total of 30 patients showed no statistically significant difference between the groups in terms of the percentages of alveolar macrophages, lymphocytes, neutrophils, eosinophils, and epithelial cells, or in the calculated macrophage/lymphocyte and lymphocyte/neutrophil ratios (all *p* > 0.05). However, a tendency toward a higher eosinophil percentage was observed in the reproductive group (*p* = 0.082). All findings are detailed in Table 4.

BALF cellular components were compared between hospitalized and non-hospitalized groups. Analyses revealed a statistically significant lower percentage of alveolar macrophages (*p* = 0.011) and a higher percentage of eosinophils (*p* = 0.036) in the hospitalized group. Additionally, the hospitalized group showed a trend toward a higher neutrophil percentage (*p* = 0.063) and a lower lymphocyte/neutrophil ratio (*p* = 0.074); however, these differences were close to the threshold for statistical significance but did not reach definitive significance. No significant differences were found between the two groups in terms of other cellular parameters. All findings are presented in detail in Table 5.

In the sub-analysis of the data, married individuals had markedly elevated BAL alveolar macrophage numbers and macrophage/lymphocyte ratios in comparison to single individuals. In addition, single individuals tended to have higher neutrophil counts (*p* = 0.054). In patients with diabetes, BAL eosinophil counts were higher, but in patients with cough complaints, they were found to be lower.

In the study group, we found no correlation between BALF cell count values and serum WBC and RFT values. However, we found a negative correlation between high CRP values and BALF alveolar macrophage values (*p* value = 0.027).

The results of bacterial, fungal, and tuberculosis cultures sent from BAL samples are summarized in Table 6. The most frequently isolated microorganism was *P. aeruginosa*, followed by *Candida* spp. (Table 6).

Patients with weight loss had significantly higher BAL lymphocyte counts and lower macrophage/lymphocyte ratios. Patients hospitalized in the past year had significantly lower alveolar macrophage counts and higher eosinophil counts compared to those who were not hospitalized. This provides a forceful signal that the BAL cell profile could be used as a prognostic marker. The presence of low macrophages and high eosinophils suggests a “high-risk” profile that may increase the risk of exacerbation and hospitalization.

## 4. Discussion

Bronchiectasis is a major health issue in patients without cystic fibrosis, and its incidence has increased with improved diagnostic methods and the widespread use of HRCT. The term “bronchiectasis” refers to a heterogeneous group of lung diseases characterized by irreversible damage and dilation of the bronchi. It has a wide range of causes and clinical presentations, varying from incidentally detected asymptomatic radiological changes to chronic sputum production and recurrent exacerbations.

The most reported etiological factor in the development of bronchiectasis is infection. However, studies in our country showed a wide variation in the rate of infections reported as the cause, ranging from 7.4% to 88.0% [11]. Other causes, including ciliary dyskinesia (3–51.3%), asthma (1.8–22%), and immunodeficiency syndromes (3–20%), follow infections. One of the most prominent effects of bacterial pathogens in the respiratory tract is their disruption of the mucociliary clearance mechanism. Commonly encountered bacteria such as *Haemophilus influenzae*, *Streptococcus pneumoniae*, and *Pseudomonas aeruginosa* slow ciliary movement and directly damage the integrity of the ciliary epithelium through various factors they secrete. In addition, they negatively affect mucus secretion and function. *H. influenzae* can trigger excessive mucus production and cause direct damage to the airway epithelium. Studies have shown that this bacterium can adhere to respiratory epithelial cells and the underlying connective tissue and invade these areas [12,13]. Our research identified microbial growth in 20 individuals (66.7%) through analysis of the BAL culture. The dominance of *Pseudomonas aeruginosa* observed in our cohort is consistent with the current literature on non-cystic fibrosis bronchiectasis, where *P. aeruginosa* has been repeatedly associated with more severe disease phenotypes, increased airway inflammation, and worse clinical outcomes. Large observational cohorts and registry-based studies have shown that *P. aeruginosa* colonization is associated with higher exacerbation rates, accelerated decline in lung function, and increased healthcare utilization in patients with bronchiectasis [14,15]. Additionally, the isolation of Enterobacteriaceae species (including *Escherichia coli*, *Klebsiella* spp., and *Enterobacter* spp.) and less frequently encountered organisms such as Stenotrophomonas maltophilia and *Pandoraea* spp. reflects an increasingly recognized, expanding, and heterogeneous airway microbiome in advanced or repeatedly treated bronchiectasis [16,17]. The presence of mixed and atypical bacterial profiles in BAL cultures highlights the clinical significance of lower airway sampling in non-CF bronchiectasis, as sputum cultures alone may underestimate microbial diversity and fail to detect potential pathogenic organisms contributing to chronic airway inflammation and disease progression [18].

Bronchiectasis is a chronic lung disease that results from an inflammatory response triggered by recurrent infections and persistent inflammation, leading to abnormal airway remodeling and structural damage [19]. There is a balance between proinflammatory and anti-inflammatory mechanisms in the body, and in patients with bronchiectasis, there is a shift towards these proinflammatory mechanisms. Neutrophils are thought to play a central role in the pathogenesis of the resulting tissue damage [20]. An increase in blood neutrophils, correlated with disease severity and bacterial colonization, has been reported in patients [9]. Neutrophils constitute the predominant cell population in sputum and BAL samples in bronchiectasis and are also found in high numbers in the lamina propria of the bronchial mucosa. Elastase, metalloproteinases, and reactive oxygen species released by activated neutrophils contribute to the weakening of the bronchial wall and the development of bronchial dilatation by degrading elastin, collagen, and proteoglycans. Neutrophil elastases can also cause epithelial damage, goblet cell hyperplasia, and mucus hypersecretion. The bronchial wall is also infiltrated by macrophages and lymphocytes; macrophages support neutrophil migration and contribute to protease release, while lymphocytes are associated with increased immunoglobulin production and immune complex formation. To a lesser extent, eosinophils and epithelial cells may also contribute to this inflammatory process [9,10,21,22,23]. The neutrophil–lymphocyte ratio is used as a biological marker of inflammation. It is calculated by dividing the number of neutrophils in a peripheral blood sample by the number of lymphocytes [10]. In our study, this ratio was calculated in BALF.

Compared to single individuals, married individuals had significantly higher BAL alveolar macrophage counts and macrophage/lymphocyte ratios, while single individuals tended to have higher neutrophil counts. The GOLD 2025 report formally recommends the patient–partner co-management model for the management of chronic respiratory diseases, emphasizing that the partner’s active participation in respiratory exercises, support for medication adherence, and active role in clinical visits are associated with increased treatment adherence, improved health-related quality of life, and measurable gains in functional capacity [24]. In another study, it was shown that the patient–partner/caregiver relationships in patients with idiopathic pulmonary fibrosis contributed to earlier detection of disease progression or acute exacerbations by enabling closer monitoring of symptoms and positively impacted the patient experience by supporting adherence to pharmacological and non-pharmacological treatments. Additionally, qualitative and observational studies indicate that regular caregiver involvement reduces the psychosocial burden, strengthens coping strategies, and leads to significant improvements in perceived health status and overall quality of life for IPF patients [25]. It is well-known that BALF results are affected by infection, inflammation, and structural lung diseases. With this study, we showed for the first time in the literature that being a partner in non-cystic bronchiectasis positively affects lung microbiota, as evidenced by changes in BALF cell composition.

BAL eosinophil counts were higher in patients with diabetes but lower in patients with cough complaints. Our findings suggest that diabetes may be associated with an unusual pattern of eosinophilic inflammation in the airways of patients with bronchiectasis. Conversely, the low eosinophil count in patients with cough symptoms suggests that cough is predominantly related to neutrophilic inflammation or mechanical factors. This paradox may point to different endotypes of bronchiectasis currently discussed in asthma and other diseases. In the literature, to investigate the anti-inflammatory efficacy of macrolide antibiotics in bronchiectasis cases, seventeen bronchiectasis cases were randomized to receive clarithromycin and supportive treatment for three months at the Department of Pediatrics of a university hospital, and the results were compared with a control group. This study showed a significant increase in BALF neutrophil count in this patient group [25].

### Limitations

The limitations include the study being single-centered, the relatively small number of patients, and the fact that BAL results may be influenced by infection, inflammation, smoking, and structural lung diseases.

## 5. Conclusions

This study demonstrates that BAL is a valuable tool beyond routine microbiological investigations for understanding the heterogeneous nature of bronchiectasis and identifying clinically relevant subgroups. Our findings reveal that the BAL cellular profile has wide-ranging clinical significance, from its relationship with weight loss, a systemic indicator of disease activity, to its potential to predict important clinical outcomes such as hospitalization. Our finding that a low alveolar macrophage and high eosinophil percentage may constitute a “high-risk” profile points to the potential use of BAL as a prognostic biomarker. Even more surprising is that a sociodemographic factor such as marital status also shows a significant relationship with the balance of immune cells in the airway microenvironment. These findings may provide an additional perspective on the pathogenesis of recurrent inflammation in patients with bronchiectasis. It is believed that receiving support from a partner may influence the alveolar microbiota. In conclusion, this hypothesis-generating study supports the notion that BAL cellular analysis is a powerful tool that could pave the way for developing personalized treatment strategies and better understanding the course of bronchiectasis. Extensive, prospective, longitudinal studies are required to elucidate the etiology and clinical relevance of these associations.

## Figures and Tables

**Table 1:** The clinical manifestations of patients.

Previous lung infection	24 (80%)
Dyspnea	20 (66.7%)
Cough	25 (83.3%)
Phlegm	29 (96.7%)
Chest pain	11 (36.7%)
Hemoptysis	10 (33.3%)
Weight loss	12 (40%)
Rhonchus	4 (13.3%)
Clubbing	13 (43.3%)
Squawk	6 (20%)

**Table 2:** Chest CT findings and bronchiectasis scores.

Reticulation	4 (13.3%)
Ground-glass opacity	6 (20%)
Consolidation	8 (26.7%)
Tubular bronchiectasis	10 (33.3%)
Cystic bronchiectasis	19 (63.3%)
Traction bronchiectasis	17 (56.7%)
FACED median (min-max)	2 (1–2)
BSI median (min-max)	9 (0–9)

FACED: FEV1, Age, Colonization with *P. aureginosa*, Radiological Extension of bronchiectasis, Modified MMR Dyspnea Score. BSI: Bronchiectasis Severity Index.

**Table 3:** The results of laboratory tests and pulmonary function tests.

Tests	Median (Min–Max)	Number of Patients with Abnormal Results (%)
White blood cells (μL)	8710 (5390–14,380)	11 (36.66%) elevated
Neutrophils (μL)	5345 (2820–11,430)	9 (30%) elevated
Lymphocytes (μL)	2140 (460–5080)	6 (20%) elevated
Hemoglobin (g/dL)	13.35 (8–16.3)	16 (53.3%) anemia
Urea (mg/dL)	26 (15–57)	6 (20%) elevated
Creatinine (mg/dL)	0.71 (0.54–1.08)	All creatinine normal
AST (U/L)	16.5 (7–85)	2 (6.6%) elevated
ALT (U/L)	18 (6–82)	1 (3.3%) elevated
CRP (mg/L)	9.85 (1–131)	23 (76.70%) elevated
FVC (%)	72 (25–108)	21 obstructive (70%)
FEV_1_ (%)	68 (18–110)	21 obstructive (70%)
FEV_1_/FVC (%)	76 (39–92)	21 obstructive (70%)

AST: Aspartate transferase. ALT: Alanine aminotransferase. CRP: C-reactive protein. FVC: Forced vital capacity. FEV_1_: Forced expiratory volume in one second.

**Table 4:** BALF culture growth and cellular composition.

BALF Cells	Total (*n* = 30) Median (Min–Max)	Culture Positive (*n* = 20) Median (Min–Max)	Culture Negative (*n* = 10) Median (Min–Max)	*p*-Value
Alveolar Macrophages	29 (2–80)	26.5 (2–70)	37.5(10–80)	0.224
Lymphocytes	10 (1–50)	10 (1–40)	19.5 (2–50)	0.100
Macrophages/Lymphocytes	2 (0.17–40)	2 (0.17–14)	1.93 (0.2–40)	0.965
Neutrophils	10 (1–90)	33.5 (1–90)	22.5(2–80)	0.140
Lymphocytes/Neutrophils	0.45 (0.01–10)	0.34 (0.01–10)	0.83 (0.04–5)	0.179
Eosinophils	2 (1–47)	5 (1–47)	1 (1–26)	0.082
Epithelial Cells	5 (0–35)	6.5 (1–35)	2(0–30)	0.220

**Table 5:** The relationship between hospital admissions and blood cell counts over the past year.

BALF Cells	Hospitalized (*n* = 21)	Non-Hospitalized (*n* = 9)	*p*-Value
	Median (Min–Max)	Median (Min–Max)	
Alveolar Macrophages	20 (2–70)	47 (10–80)	0.011
Lymphocytes	10 (1–40)	10 (2–50)	0.697
Macrophages/Lymphocytes	1.87 (0.17–14)	3.5 (0.20–40)	0.094
Neutrophils	32 (4–90)	20 (1–80)	0.063
Lymphocytes/Neutrophils	0.37 (0.01–7.75)	1 (0.13–10)	0.074
Eosinophils	5 (1–47)	1 (1–5)	0.036
Epithelial Cells	5 (0–35)	3 (1–25)	0.963

**Table 6:** Culture results.

Microorganism (*n* = 20)	*n* (%)	Antibiotic Resistance Status (*n*)
*Pseudomonas aeruginosa*	8 (26.66%)	MDR (8)
*Klebsiella* spp.	3 (10%)	XDR (2)
		MDR (1)
*Candida* spp.	3 (10%)	-
*Methicillin-Sensitive Staphylococcus aureus* (*MSSA*)	1 (3.33%)	MSSA
*Stenotrophomonas maltophilia*	1 (3.33%)	-
*Pandoraea* spp.	1 (3.33%)	-
*E. coli*	1 (3.33%)	MDR
*Enterobacter* spp.	1 (3.33%)	MDR
*Beta-Hemolytic Streptococcus*	1 (3.33%)	-

## Data Availability

Data is available upon scientific request to the corresponding author due to local regulations restricting patients’ data.

## Abbreviations

AMs	Alveolar Macrophages
AST	Aspartate Transferase
ALT	Alanine Aminotransferase
BAL	Bronchoalveolar Lavage
BALF	Bronchoalveolar Lavage Fluid
BSI	Bronchiectasis Severity Index
CRP	C-Reactive Protein
FACED	FEV1, Age, Colonization of *P. aureginosa*, Radiological Extension of Bronchiectasis, Modified MMR Dyspnea Score
FEV_1_	Forced Expiratory Volume in One Second
FVC	Force Vital Capacity
PMNs	Polymorphonuclear Neutrophils
RFT	Respiratory Function Test
